# On Your Own: An Explorative Study on the Psychopathological and Psychosocial Correlates of Hikikomori Symptoms in Dutch Adolescents and Young Adults

**DOI:** 10.1007/s10578-025-01828-0

**Published:** 2025-03-18

**Authors:** Peter Muris, Annelie van der Veen, Britney Luijten, Charlotte de Bie, Cor Meesters

**Affiliations:** 1 Faculty of Psychology and Neuroscience, Department of Clinical Psychological Science, Maastricht University, P.O. Box 616, Maastricht, 6200 MD The Netherlands; 2 Stellenbosch University, Stellenbosch, South Africa; 3 Youz Parnassia Group, Maastricht, The Netherlands

**Keywords:** Hikikomori, Extreme social withdrawal, Psychopathology, Loneliness and aloneness, Problematic internet use, Autism spectrum disorder

## Abstract

Hikikomori is an extreme form of social withdrawal that has been originally described in Japan but that is emergent in many other developed and industrialized countries. The present exploratory study was conducted in The Netherlands and aimed to examine psychopathological and psychosocial correlates of hikikomori symptoms as measured with the Hikikomori Questionnaire-25 (HQ-25) in a convenience population of 122 non-clinical adolescents and young adults and 24 clinically referred young people who were suspected to suffer from autism spectrum disorder (ASD; of which 75% eventually received this DSM-5 diagnosis). The results indicated that higher levels of hikikomori symptoms were associated with higher levels of psychopathological risk factors, higher levels of loneliness as well as a more positive attitude towards being alone, and higher levels of problematic internet use. Furthermore, it was found that hikikomori symptoms were significantly higher in clinically referred participants with suspect ASD than in non-clinical participants. It can be concluded that hikikomori symptoms were present in this sample of young Dutch participants and showed theoretically meaningful relations with other psychological constructs.

## Introduction

Social withdrawal refers to the tendency to retreat oneself from social interactions and relationships [1]. It is an umbrella phenomenon that may be guided by various underlying processes. Asendorpf [2] used the concepts of social approach and social avoidance to discern three basic types of withdrawal. The *unsociable* or *uninterested* type is characterized by low social approach and low social avoidance and is concerned with individuals who seek solitude because they have less interest in other people and like to be on their own. The *avoidant* type is typified by low social approach and high social avoidance and pertains to persons who prefer to evade social interactions because these are experienced as stressful and challenging. The *shy* type, finally, is specified by high social approach and high social avoidance and refers to people who want to be socially involved but at the same time lack the confidence to do so and hence engage with restraint in interactions with others. Although research has indicated that Asendorpf’s account suffers from various limitations [3, 4] – showing that besides social motivations other potential mechanisms are implicated in social withdrawal, the model has been very influential prompting many scholars to understand that withdrawal behavior may arise from many different reasons, which in turn have different implications for people’s socioemotional functioning and well-being [5]. Social withdrawal can occur throughout all stages of life but appears to be particularly prominent during the developmental stages of adolescence and early adulthood [6] when new social ties have to be formed in extrafamiliar contexts under (partly biologically mediated) social-evaluative pressures [7, 8].

Hikikomori is generally seen as a severe form of social withdrawal, characterized by extreme social isolation and seclusion for a longer period-of-time (6 months or more) and a disengagement from the responsibilities of contemporary life such as following education or committing to work [9]. Originally considered as a syndrome that would only occur in Japan [10] and listed as ‘cultural concept of distress’ in the latest edition of the Diagnostic and Statistical Manual of Mental Disorders (DSM-5) [11], there is increasing evidence that hikikomori is a global phenomenon that appears to be present in many developed and industrialized Western and Eastern countries [12], including the Netherlands [13] where the current study was conducted. Evidence on the prevalence of hikikomori has mainly been gathered in Japan where it is estimated that up to 2% of the population lives this type of hermit-like existence [14, 15, 16], but a recent study has reported a highly similar prevalence rate of severe social isolation – as a proxy of hikikomori – in a representative sample of the population in 29 European countries [17]. Note that although the hikikomori syndrome refers to a complete seclusion in one’s house/bedroom without having any contact with other (cohabiting) persons, there are also milder forms, for example persons who display acts of social isolation while occasionally meeting others outside of the house [18]. This underlines the dimensional nature of social withdrawal and hikikomori and indicates that subclinical manifestations of these phenomena can also be found in the general population.

Evidence clearly indicates that social withdrawal and isolation and its extreme variant of hikikomori are indicative for the presence of psychopathology [19]. Social anxiety disorder and depressive disorder are the two most prominent examples of mental disorders that have been associated with the tendency to socially seclude and isolate oneself [20]. In social anxiety disorder, the fear of being judged or scrutinized by others prompts the person to avoid meeting (new) people or attending social gatherings [21], whereas in depressive disorder social withdrawal may be fueled by social anhedonia – an increased disinterest in interactions with others and a lack of pleasure in social situations [22].

Furthermore, in recent years, support has also been found for a link between autism spectrum disorder (ASD) and hikikomori. For example, Katsuki et al. [23] compared a group of hikikomori patients (*n* = 103) and a group of clinical control patients without hikikomori (*n* = 221) with regard to their scores on the Autism-spectrum Quotient, a self-report scale for measuring autistic traits [24]. It was found that the hikikomori patients displayed significantly higher levels of autistic traits – in particular social skills and communication deficits and poor imagination (but not exceptional attention to detail and poor attention control) – as compared to the clinical control patients. Cross-sectional surveys administered in non-clinical adults have confirmed the positive relation between autistic traits and hikikomori symptoms [25, 26]. Up until now, there is only one study that examined the hikikomori phenomenon from the perspective of ASD patients. In that study, Yamada et al. [27] conducted interviews with 39 clinically referred, adult ASD patients to explore their symptoms of social withdrawal. The results indicated that a substantial proportion of the patients with ASD (41.0%) showed severe social withdrawal symptoms and could be qualified as hikikomori. Although it is possible be that the link between ASD and hikikomori is a measurement artifact caused by shared symptoms occurring in both conditions, it seems likely that ASD really conveys a risk for developing hikikomori because the social deficits and social burden associated with this neurodevelopmental problem could lead to extreme levels of social withdrawal [23].

Social withdrawal and hikikomori are frequently linked to loneliness [28], which has been defined as a sense of sadness and emptiness that arises when people perceive that the quantity and/or quality of their social relationships is insufficient [29]. For example, in a sample of 36 individuals with hikikomori from India, Japan, Korea, and the United States, Teo et al. [30] found a mean score of 55 on the UCLA loneliness scale [31], which appeared to be far above the average score as documented in the normal population from these countries. Some scholars converge on the notion that prolonged periods of social isolation result in increased feelings of loneliness [18, 32], but there are also researchers who have postulated a reversed scenario in which social disconnection and loneliness promote social withdrawal and hikikomori symptoms [33]. Whatever the case may be, there is little doubt about the fact that loneliness is a prominent feature in those who withdraw and isolate themselves [34]. Meanwhile, it has also been noted that some individuals show a tendency to withdraw themselves because they have a stronger preference for solitude [35]: they desire to be alone in order to engage in a favorite activity, to escape from an overstimulating environment, or to contemplate on some aspect of life [36]. Thus, it is good to be aware of the fact that people who display extreme social withdrawal may be trapped in what has been labelled as the loneliness-solitude paradox [37]: on the one hand these individuals wish to be on their own, but on the other hand they feel sad because they have so little contact with others.

Various studies have revealed a significant relation between hikikomori and the (excessive) use of the internet [38]. More specifically, individuals who display extreme social withdrawal show higher levels of internet addiction symptoms [6, 39, 40, 41] and problematic smartphone use [40], and more frequently display excessive online gaming behavior [26, 42, 43, 44]. An analysis of the evidence suggests that the relationship between excessive internet use and social withdrawal behavior is reciprocal [45]. That is, the excessive employment of the internet likely undermines and diminishes people’s social behavior and as such promotes withdrawal tendencies. Meanwhile, it is also plausible that the frequent employment of internet is a phenomenon occurring in people who for some reason are socially isolated. It is still largely unclear what exact motives underlie the (increased) internet use of withdrawn or hikikomori persons [46, 47]. Online activities might be a good way to pass time by seeking entertainment, provide an opportunity to socialize with others, or offer an escape from the problems encountered in life, but obviously this topic requires further investigation.

The present study is an exploratory investigation of the hikikomori phenomenon in The Netherlands and aims to examine the correlates of hikikomori symptoms – as measured with a Dutch translation of the widely used Hikikomori Questionnaire-25 (HQ-25) [48]. A survey was construed that was first of all administered in a convenience sample of 122 non-clinical adolescents and young adults from the Netherlands to investigate the relations between hikikomori symptoms and (a) psychopathological symptoms that are thought to convey a risk for developing extreme social withdrawal, which were measured with the Hikikomori Risk Index-24 (HRI-24) [49], (b) feelings of loneliness and attitudes towards being alone, and (c) levels of problematic internet use and various motives for going online. In addition, to further explore the relationship between hikikomori symptoms and (d) ASD, we ad hoc collected similar data in a small clinical sample of young people (*n* = 24) suspected of having this neurodevelopmental disorder. It was hypothesized that (1) there would be positive associations between hikikomori symptoms and various types of psychopathological risk factors, (2) hikikomori symptoms would be positively correlated with loneliness as well as a positive attitude towards being alone, (3) hikikomori symptoms would be positively associated with levels of problematic internet use and social, coping, and enhancement motives for going online, and (4) hikikomori symptoms would be higher in clinically referred participants with suspect ASD than in the non-clinical participants. While the study aimed to replicate a number of findings that have already been obtained in previous research, it is important to note that there are also a number of new elements such as the relationship between the HQ-25 and HRI-24 (which surprisingly has not been studied so far), the link between hikikomori symptoms and aloneness, and the relations between hikikomori symptoms and motives for internet use. Furthermore, while Yamada et al. [27] compared groups of ASD patients with and without hikikomori, the present study was the first to contrast levels of hikikomori symptoms between clinically referred patients with ASD or high autistic traits and non-clinical participants.

## Method

### Participants and Procedure

The non-clinical sample consisted of 122 adolescents and young adults (82 females and 38 males, 2 participants did not report their sex) who were living in Limburg, the southern part of the Netherlands. The participants ranged in age between 16 and 27 years (mean age = 19.61 years, *SD* = 3.09). Half of them were recruited in secondary schools (*n* = 61) and were following either pre-vocational secondary education (*n* = 9), senior general secondary education (*n* = 18), or pre-university education (*n* = 33); one student had dropped out and was no longer in school. The other half was recruited via snowball sampling in the network of the researchers (AvdV, BL, CdB) and these participants were in different types of tertiary education including vocational education (*n* = 6), higher professional education (*n* = 11), or university (*n* = 44).

Participants were invited by email or via social media to take part in an online survey study on correlates of social withdrawal in the Spring (April-June) of 2024. The invitation contained a link to Qualtrix, the online survey platform used by Maastricht University. Participants were first guided to an information letter that described the purpose of the study, its methodology, potential risks and benefits, privacy information, and the contact details of the researchers, after which they could confirm their participation on an online consent form. Following this, they were directed to the survey, which consisted of some questions about demographics (i.e., age, gender, and educational level) and five standardized questionnaires (see below). When participants had finished, they received a brief debriefing letter with a more detailed description of the aims of the study and a verbal acknowledgement for their participation. Initially, 213 participants gave their consent and started with the online survey. However, 91 of them had to be discarded because they did not finish their survey, yielding a response rate in the non-clinical sample of 57.3%.

The procedure in the clinical sample was somewhat different. The 24 participants (12 females and 12 males) were patients of an outpatient mental health clinic (Youz Parnassia Group) in Maastricht, The Netherlands. All patients were seen for a psychological assessment between November 2023 and December 2024 by the first author who worked at this clinic as a licensed psychologist. If the target of the psychological evaluation was to find out whether a diagnosis of ASD was applicable, patients were verbally invited by the psychologist-researcher whether they were willing to participate in the study at the end of the assessment session. If they indicated interest in participating, they received the information letter and consent form. After providing their informed consent, they received and completed a shortened paper-and-pencil version of the survey (the scales for measuring problematic internet use and motives for going online were not administered in the clinical sample). Of 26 consecutively approached patients only two were not willing to participate, resulting in a response percentage of 92.3%.

The mean age of the clinical participants was 16.63 years (*SD* = 2.41, range 13–21 years) and most of them were still in secondary education (*n* = 19), following pre-vocational secondary education (*n* = 5), senior general secondary education (*n* = 6), or pre-university education (*n* = 8). The others were in tertiary education: four followed vocational education and one was in university. The majority of the patients were indeed diagnosed with ASD (*n* = 18, 75.0%) after an assessment procedure that included clinical observations, the Autism Diagnostic Interview [50], the Autism Diagnostic Observation Schedule [51], and other assessment tools relevant for ASD (e.g., theory-of-mind and faux-pas tests). The other six patients (25.0%) – although high on autistic traits – did not receive a formal DSM-5 diagnosis of ASD. Comorbid problems were frequently present in the patients: nine of them were also diagnosed with attention-deficit/hyperactivity disorder, 15 displayed emotional psychopathology (e.g., depression, social anxiety disorder, eating disorder, obsessive-compulsive disorder), while 14 had clear schooling problems (e.g., truancy, school refusal, frequent school absenteeism).

It should be noted that the non-clinical and clinical samples were found to be quite dissimilar in terms of sex [χ(1) = 3.17, *p* =.075], age [t(144) = 5.28, *p* < .001], and educational level [χ(1) = 5.44, *p* <.05]: the non-clinical sample tended to contain relatively more females (68.9% versus 50.0%), was older (mean ages in years being 19.61, *SD* = 3.09 versus 16.63, *SD* = 2.41), and more often followed education at a (pre-) university level (63.1% versus 37.5%).

### Measures

**Hikikomori Questionnaire-25** (HQ-25) [48]. The HQ-25 measures hikikomori symptoms and consists of 25 items that can be allocated to the prototypical signs of diminished socialization (11 items; e.g., “I stay away from other people”, “I strongly prefer to be around other people” [reversed item]), isolation (8 items; e.g., “I spend most of my time at home”, “I shut myself in my room”), and perceived lack of emotional support (6 items; e.g., “There really is not anyone with whom I can discuss matters of importance”, “There are people in my life who try to understand me” [reversed item]). Each item has to be rated on a 5-point Likert scale with 0 = strongly disagree, 1 = somewhat disagree, 2 = neither agree nor disagree, 3 = somewhat agree, and 4 = strongly agree. HQ-25 total and subscale scores can be calculated by summing the ratings of relevant items (after recoding the ratings on reversed items). The initial psychometric evaluation of the HQ-25 conducted in a Japanese population has indicated that the scale has a clear three-factor structure, is reliable in terms of internal consistency (with a Cronbach’s alpha of 0.96 for the total score) and test-retest stability (the test-retest correlation was 0.92), while evidence was also found for the convergent (through positive correlations with loneliness and a preference for solitude) and discriminant validity (scores on the scale differed between participants with and without hikikomori) of the scale [48]. Studies in other countries have yielded highly similar results in adult as well as adolescent populations [52, 53, 54, 55, 56, 57, 58], making the HQ-25 the most widely used scale for measuring hikikomori symptoms (see www.dralanteo.com). The original study by Teo et al. [48] established a cut-off score of 42 that can be used to identify individuals with a liability for developing extreme social withdrawal. In the current study, the Dutch version of the HQ-25 was used, which was created following a translation–back-translation method conducted by the first author and a certified translator.

**Hikikomori Risk Index-24** (HRI-24) [49]. This self-report scale assesses symptomatology of psychopathological conditions that might increase the risk for hikikomori and can be used in adults as well as adolescents. More specifically, 24 items measure symptoms of (a) anthropophobia (4 items; i.e., fear of people; e.g., “I am uncomfortable when I am in touch with others); (b) agoraphobia (4 items; e.g., “I am afraid of being in crowded places”); (c) paranoia (7 items; e.g., “Confiding with others is dangerous because people might then reveal my secrets”); (d) lethargy (4 items; e.g., “I often feel weak and lacking energy”); and (e) depressive mood (5 items; “I feel a sense of inner emptiness”). Participants have to indicate on a 5-point Likert scale how much each item is applicable to them with 1 = strongly disagree and 5 = strongly agree. By combining relevant items, a subscale score can be obtained for each type of psychopathology. The original study by Loscalzo et al. [49] demonstrated that the subscales of HRI-24 are internally consistent (with Cronbach’s alphas varying between .82 and .91). Furthermore, confirmatory factor analysis yielded a good fit for the hypothesized correlated five-factor structure of the scale. Finally, HRI-24 scores correlated positively with an alternative index of extreme social withdrawal risk (i.e., the NEET-Hikikomori Risk factors scale) [59]. Similar favorable psychometric qualities have been noted in other psychometric evaluations of the HRI-24 [60, 61].

**Loneliness and Aloneness scale for Children and Adolescents** (LACA; in Dutch known as the Leuvense Eenzaamheidsschaal voor Kinderen en Adolescenten) [62]. The LACA comprises four scales of 12 items each that measure: (a) loneliness in relationships with parents (e.g., “I feel I have very strong ties with my parents” [reversed item]); loneliness in relationship with peers (e.g., “I think I have fewer friends than others”); aversion to aloneness (e.g., “When I am alone, I feel bored”); and affinity for aloneness (e.g., “I want to be alone”). Items are rated on a 4-point Likert scale with labels ‘often’, ‘sometimes’, ‘rarely’, and ‘never’, and these are scored 4, 3, 2, and 1, respectively, with the exception of nine items included in the ‘loneliness in relationships with parents’ scale, for which scores have to be reversed. Psychometric evaluation has shown that the LACA has excellent reliability (with all Cronbach’s alphas >.80), good construct validity (i.e., data strongly converge with the hypothesized four-factor structure), and concurrent validity (as evidenced by the expected correlations with other measures of loneliness and solitude in young people) [63, 64]. The LACA has been developed and validated in youth samples [62] but since there was no alternative measure effectively combining the constructs of loneliness and aloneness, we decided to use the scale in the present sample of adolescents and young adults.

**Problematic Internet Use Scale for Adolescents** (PIUS-A) [65]. The PIUS-A is a brief and simple 11-item measure that can be used to screen for problematic internet use in young people aged 11 years and older. It was developed on the basis of a thorough review of the instruments that have been previously proposed by other scholars. The PIUS-A contains items such as “I’ve sometimes tried to control or reduce my internet use, but I couldn’t”, “I sometimes get irritated or in a bad mood because I can’t connect to the internet or because I have to disconnect”, and “I’ve sometimes got into trouble because of the internet” that have to be rated on a 5-point scale with 0 indicating ‘totally disagree’ and 4 indicating ‘totally agree’. A total score can be calculated by summing across all items, with higher scores reflecting higher levels of problematic internet use. Factor analysis has revealed that the PIUS-A is a one-dimensional measure with satisfactory internal consistency (Cronbach’s alpha = 0.82) and good potential to discriminate between youth with and without excessive internet use [65]. Other studies have also demonstrated the usefulness of the PIUS-A as a screening index for problematic internet use [66] that correlates in a theoretically way with other relevant clinical constructs [67].

**Motives for Using the Internet scale** (MUI) [68]. This scale was specifically developed to understand why people employ the internet and continue to stay online despite a range of negative outcomes. The scale consists of 20 items that can be allocated to five subscales of four items each that represent the following motives for using the internet: (a) utility, which is concerned with using the instrument for practical reasons (e.g., “I use the internet to obtain information”); (b) enhancement, which means that the person wants to create a positive emotional state by going online (e.g., “I use the internet because it is fun”); (c) social, which implies that the person uses the internet to get in touch with other people (e.g., “I use the internet to meet new people”; (d) coping, which refers to attempts to connect to the internet because one tries to avoid negative feelings and distress (e.g., “I use the internet because it helps me to feel better when I am nervous”); and (e) conformity, which pertains to the person going online to avoid the disapproval from others (e.g., “I use the internet to not feel excluded by others”). Respondents are asked to rate the applicability of each item using a 5-point Likert scale with 1 = never, 2 = rarely, 3 = half of the time, 4 = most of the time, and 5 = always. Scores can be combined to yield subscale scores for each of the motives. Various studies have indicated that the internal consistency coefficients of various MUI subscales are good, that the scale indeed possesses the hypothesized 5-factor structure and shows the to be expected pattern of associations with indices of problematic internet use [68, 69, 70]. So far, the scale has only been used in adult populations, but the items were considered simple and clear enough to be also completed by the younger participants in our sample.

### Statistical Analyses

The Statistical Package for Social Sciences (SPSS) was used to analyze the data. Apart from some basic statistics used to calculate means, standard deviations, and Cronbach’s alpha coefficients for various measures, the first three hypotheses were examined by means of Pearson correlations and regression analyses. In the regression analyses, which were conducted to control for shared variance among (predictor) variables and thus enabled us to identify unique correlates of hikikomori symptoms, HQ-25 scores were always the dependent variable while psychopathological symptoms (HRI-24), loneliness and attitudes towards aloneness (LACA), and motives for using the internet (MUI) were the predictor variables. Regression analyses were controlled for demographic variables because these were significantly related to some psychological constructs. For example, gender had an effect on MUI enhancement (i.e., females more often indicated to use the internet for creating a positive emotional state than males; *t*(120) = 1.95, *p* = .05, Cohen’s *d* = 0.37), while age and educational level were negatively correlated with HQ-25 hikikomori symptoms (*r*’s being − 0.46 and − 0.26, *p’s* < 0.01) as well as with various types of psychopathological risk factors and motives of internet use (*r*’s between − 0.19 and − 0.41, all *p*’s < 0.05). Collinearity statistics were inspected for all regression analyses and indicated that predictor variables were not too strongly correlated (all VIF values < 4 and all Tolerance values > 0.25) [71]. To compare the HQ-25 scores of non-clinical and clinically referred participants with suspect ASD (hypothesis 4), a univariate analysis of variance (ANOVA) was conducted. Because both samples were different in terms of age, educational level, and sex, these demographic variables were included as covariates (i.e., ANCOVA).

## Results

### General Findings: Factor Structure and Reliability of the HQ-25

Before addressing the main hypotheses of the present study, some general findings are discussed. First, although the sample size was rather small, we conducted a confirmatory factor analysis of the Dutch version of the HQ-25 using the AMOS package of SPSS. The hypothesized model with three correlated factors referring to symptoms of diminished socialization, isolation, and lack of emotional support showed poor fit indices (NFI = 0.71, TLI = 0.77, IFI = 0.79, CFI = 0.79, χ^2^/df = 2.70, RMSEA = 0.11). Further inspection of the three-factor model by means of exploratory factor analysis revealed one strong factor of 15 items essentially combining symptoms of diminished socialization and isolation (eigenvalue = 10.67, 42.70%) and two smaller factors: a (method) factor consisting of five reversed items (eigenvalue = 2.01, 8.05%) and a factor of five items representing lack of emotional support symptoms (eigenvalue = 1.63, 6.53%). Given these results it was decided to only use the HQ-25 total score as a general index of hikikomori symptoms and not to rely on the inconclusive factor/subscale scores. Second, the internal consistency reliability of the HQ-25 total score was good (Cronbach’s alpha = 0.93). Satisfactory internal consistency coefficients were also found for most other (sub)scales (see Table 1); the only notable exception was the MUI utility subscale, which had an unsatisfactory alpha of 0.56.

**Table 1:** Mean scores and reliability coefficients for various measures as obtained in the non-clinical sample, and pearson correlations and regression coefficients showing the (unique) associations between Hikikomori symptoms as indexed by the HQ-25 and other questionnaires

	M (SD)	Cronbach’s $\:\alpha$	*r* HQ-25	β^†^ HQ-25
HQ-25				
Hikikomori symptoms	25.14 (16.01)	0.93		
HRI-24				
Anthropophobia	6.90 (3.21)	0.86	0.78***	0.44***
Agoraphobia	6.60 (3.28)	0.82	0.68***	0.12
Paranoia	13.47 (5.13)	0.84	0.66***	0.13
Lethargy	7.62 (3.37)	0.80	0.62***	0.00
Depressive mood	8.58 (4.13)	0.87	0.65***	0.17*
LACA				
Loneliness parents	18.86 (7.12)	0.93	0.41***	0.15*
Loneliness peers	21.91 (7.47)	0.91	0.53***	0.49***
Aloneness aversion	27.98 (6.35)	0.86	− 0.13	− 0.34***
Aloneness affinity	33.63 (5.61)	0.84	0.44***	0.21**
PIUS-A				
Problematic internet use	11.95 (7.70)	0.84	0.46***	
MUI				
Utility	11.43 (2.67)	0.56	− 0.18*	− 0.14
Enhancement	13.11 (2.85)	0.83	0.00	− 0.12
Social	6.48 (2.65)	0.82	0.40***	0.11
Coping	8.46 (3.43)	0.89	0.33***	0.26*
Conformity	8.00 (2.62)	0.68	0.22*	− 0.01

Note. *N* = 122. HQ-25 = Hikikomori Questionnaire-25, HRI-24 = Hikikomori Risk Index-24, LACA = Loneliness and Aloneness scale for Children and Adolescents, PIUS = Problematic Internet Use Scale for Adolescents, MUI = Motive for Using the Internet scale. ^†^Results of linear regression analysis, method enter (also controlling for age, sex, and educational level), conducted for each questionnaire separately

*p < .05, ***p < .001. For clarity, statistically significant effects are printed in bold

### Hikikomori Symptoms and Psychopathological Risk Symptoms

As shown in Table 1, correlations between HQ-25 scores and various types of psychopathological risk indicators as indexed by the HRI-24 were all positive, strong, and statistically significant (i.e., *r*’s being 0.62 or larger, all *p*’s < 0.001). This indicates that higher levels of hikikomori symptoms were associated with higher levels of psychopathological symptoms of anthropophobia, agoraphobia, lethargy, paranoia, and depressive mood.

A linear regression analysis was conducted predicting HQ-25 scores from the demographic (control) variables of sex, age, educational level (block 1) and HRI-24 subscales scores (block 2). The results indicated that the demographic variables accounted for a significant proportion of the variance [22.9%; *F*(3,118) = 11.66, *p* < .001], with age being the only statistically significant unique predictor (β = − 0.45, *p* < .001). The HRI-24 subscales that were entered in block 2 accounted for an extra 47.7% of the variance in HQ-25 scores [*F*(8,113) = 33.92, *p* < .001]. It was found that symptoms of anthropophobia (β = 0.44, *p* < .001) and to a lesser extent depressive mood (β = 0.17, *p* < .05) made significant unique contributions to hikikomori symptoms.

### Hikikomori Symptoms and Feelings of Loneliness and Attitudes Towards Being Alone

HQ-25 scores were positively correlated with the LACA loneliness in relationships with parents, loneliness in relationships with peers, and an affinity for aloneness (*r*’s between.41 and 0.53, all *p*’s < 0.001; see Table 1). Thus, higher levels of hikikomori symptoms were associated with stronger feelings of loneliness and a more positive attitude towards being alone. The correlation between the HQ-25 total score and LACA aversion to aloneness was not significant (*r* = −.13).

Regression analysis indicated that (after controlling for sex, age, and educational level) the LACA scales accounted for a statistically significant proportion of the variance in HQ-25 total scores [35.0%, *F*(4,114) = 23.72, *p* < .001]. All four scales appeared to make unique and statistically significant contributions to hikikomori symptoms. Positive beta values were found for loneliness in relationships with peers (which emerged as the strongest predictor), affinity for aloneness, and loneliness in relationships with parents (which made a relatively small contribution), while a negative beta value was noted for aversion to aloneness.

### Hikikomori Symptoms and Problematic Internet Use and Motives for Going Online

The mean PIUS-A score was 11.95 (*SD* = 7.70), which was comparable to the value reported by Rial Boubeta et al. [65]. Using the proposed cut-off of 17, it was found that 20.5% of the participants in our non-clinical sample showed a level of internet use that could be labelled as ‘problematic’. Inspection of the MUI subscales scores revealed that enhancement was the most frequently mentioned motive for participants to go online, followed by utility, coping/conformity, and social, which was the least endorsed motive of why participants used the internet (all paired *t*’s ≥ 5.34, *p*’s < 0.001). Positive and statistically significant positive correlations were noted between the PIUS-A total score and scores on the MUI subscales of coping (*r* =.60, *p* < .001), conformity (*r* =.52, *p* < .001), social (*r* =.48, *p* < .001), and enhancement (*r* =.31, *p* =.001), indicating that these motives were more prominent among participants with higher levels of problematic internet use.

The analyses conducted to examine the relations between HQ-25 and PIUS-A/MUI scores are displayed in Table 1. As can be seen, HQ-25 scores were positively and significantly correlated with problematic internet use as indexed by the PIUS-A (*r* =.46, *p* < .001). Thus, higher levels of hikikomori symptoms were associated with higher levels of excessive and troublesome online behaviors. Note also that the HQ-25 total score was also positively correlated with the MUI social, coping, and conformity subscales (with *r*’s between 0.22 and 0.40). In other words, higher levels of hikikomori symptoms were associated with a stronger inclination to use the internet for connecting to people, dealing with problems, and obliging to the pressures of others. Meanwhile, a negative correlation between HQ-25 and MUI utility was found (*r* = −.18), indicating that participants with higher levels of hikikomori symptoms were less likely to use the internet for practical reasons.

To explore the unique relations between hikikomori symptoms and motives for using the internet, a regression analysis was conducted with the HQ-25 total score as the dependent variable and MUI subscales as the predictors. The results indicated that (after controlling for demographic variables) MUI subscales accounted for 10.8% of the variance in hikikomori symptoms [*F*(5,113) = 3.66, *p* < .01], with the coping motive being the only statistically significant positive predictor (β = 0.26, *p* < .05). Thus, higher levels of hikikomori symptoms were associated with higher levels of using the internet as a way to deal with negative emotions and distress.

### Hikikomori Symptoms in Clinical and Non-clinical Participants

A univariate ANOVA (with sex, age, and educational level as covariates, i.e., ANCOVA) was carried out to compare the HQ-25 total score of non-clinical participants and clinically referred participants with suspect ASD. This analysis showed that clinically referred participants with ASD or high autistic traits reported statistically significantly higher levels of hikikomori symptoms as compared to the non-clinical participants [*F*1,141) = 5.71, *p* < .05, η_p_^2^ = 0.03; without covariates: *F*(1,144) = 18.03, *p* < .001, Cohen’s *d* = 0.95]. The mean scores (standard deviations) of both samples are shown in Table 2. When correcting for the effects of the covariates, the estimated marginal means were 34.74 (*SE* = 3.24) for the clinically referred participants with ASD or high levels of autistic traits versus 26.23 (*SE* = 1.36) for the non-clinical participants. Using Teo et al.’s [48] HQ-25 cut-off score of 42, it was found that close to half of the sample with suspect ASD (i.e., 45.8%) displayed a liability for developing extreme social withdrawal versus only 13.9% in the non-clinical sample [χ(1) = 13.17, *p* < .001].

**Table 2:** Levels of Hikikomori symptoms (as indexed by the HQ-25) in the non-clinical sample (*n* = 122) and the clinically referred sample of adolescents/young adults with ASD or high levels of autistic traits (*n* = 24)

	Non-clinical sample	Clinical sample
HQ-25		
Mean score (*SD*)	25.14 (16.01)	40.29 (15.83)***
Estimated marginal mean (*SE*)^†^	26.23 (1.36)	34.74 (3.24)*
Percentage > cut-off^‡^	13.9	45.8***

Note. HQ-25 = Hikikomori Questionnaire-25. ^†^Corrected for age, sex, and educational level. ^‡^HQ-25 score of 42 as suggested by Teo et al. [48]

*p < .05, ***p < .001

A number of additional ANCOVAs were conducted comparing both samples with regard to their HRI and LACA scores. It was found that clinically referred participants with ASD or high levels of autistic traits exhibited significantly higher levels of anthropophobia [*F*(1,141) = 8.84, *p* < .01, η_p_^2^ = 0.06] and paranoia symptoms [*F*(1,141) = 9.86, *p* < .01, η_p_^2^ = 0.07], and also displayed a stronger affinity for aloneness [*F*(1,141) = 12.98, *p* < .001, η_p_^2^ = 0.08] and a tendency to report higher levels of loneliness in relationships with peers [*F*(1,141) = 3.60, *p* =.06, η_p_^2^ = 0.03] as compared to non-clinical participants.

## Discussion

This cross-sectional, survey-based study examined correlates of hikikomori symptoms in a mixed population consisting of non-clinical and clinically referred adolescents and young adults from the Netherlands. A first conclusion of this research is that the Dutch translation of the HQ-25 can be reliably used to measure hikikomori symptoms in younger people of this West-European country. The Cronbach’s alpha of 0.94 was comparable to that obtained for the original scale [48]. Further, as will be discussed below, HQ-25 scores correlated in a (theoretically) meaningful way with other relevant constructs, which can be viewed as evidence for the validity of this measure. Finally, although the average HQ-25 score obtained in the present study [in the combined sample (*N* = 146): *M* = 27.63, *SD* = 16.89] was somewhat lower than in previous investigations using this instrument [48, 53], it is also clear that a substantial minority of the participants (19.2%, in most cases clinically referred participants) displayed scores above the ‘hikikomori’ cut-off as proposed by Teo et al. [48], signifying that the phenomenon of extreme social withdrawal is also present in the Netherlands [9, 17].

With regard to the relation between hikikomori and psychopathological risk factors, it can be concluded that extreme social withdrawal symptoms were positively associated with a broad range of psychological problems as measured with the HRI-24. Thus, in keeping with what has been found in previous research, higher levels of hikikomori symptoms are accompanied by higher levels of anxiety problems [72],

lethargy and depressive mood [73], and paranoia symptoms [74], all of which may prompt individuals to refrain from social engagement while seeking safe seclusion in their home. This aligns well with the notion that hikikomori constitutes the ultimate consequence of a deranged disorder (e.g., social anxiety disorder, depressive disorder, schizophrenia), but it is also plausible that strong withdrawal symptoms in turn play an important role in the continuation of psychiatric problems [20, 45, 75].

Anthropophobia appeared to be the psychopathological risk factor that was most clearly correlated with hikikomori symptoms. When controlling for the shared variance with other HRI-24 scales, this type of psychological problem emerged as a strong unique predictor of extreme social withdrawal in the regression analyses. Anthropophobia can be described as ‘fear of people’ and is characterized by symptoms such as fear of making eye contact and fear of blushing, unusual eye or facial expressions, body dysmorphia and odors [76]. It is generally seen as an Eastern culture variant of social anxiety disorder [77]. As such, this finding is nicely in keeping with previous investigations showing that symptoms of hikikomori are often present in individuals with social anxiety disorder [72] or vice versa that social anxiety symptoms are often prominent in persons who display extreme social withdrawal [19]. The unique contributions of other HRI-24 factors were much smaller (depressive mood) or even non-significant (i.e., agoraphobia, lethargy, and paranoia), which may have to do with the fact that this was a non-clinical sample, in which these types of risk factors play a less prominent role in hikikomori symptomatology.

Our hypothesis that hikikomori symptoms would be associated with higher levels of loneliness but at the same time were also accompanied by a more positive attitude towards being alone was indeed confirmed by the data. This suggests that people who demonstrate extreme social withdrawal may find themselves caught in what is known as the loneliness-solitude paradox [37]: they may actually seek and value to be on their own (for a variety of reasons), but simultaneously experience feelings of abandonment and isolation. In other words, in hikikomori individuals, the ‘need to be alone’ [78] and the ‘need to belong’ [79] seem to be at odds with each other. In the psychological literature, there is a strong tendency to depict these persons as ‘unhappy’ and ‘lonely’ [28, 30, 33], but the current findings as well as data from other studies increasingly underline that the relation between social withdrawal and emotional well-being is far more complex [34, 35]. As rightly noted by Yong [34], the relationship between hikikomori and loneliness needs to be reevaluated and should take other moderating variables (such as the preference for solitude) into account.

The relations between hikikomori symptoms and internet use variables were largely as anticipated. It was found that higher levels of hikikomori features were indeed associated with higher levels of problematic internet use [38]. It seems plausible that the use of internet-mediated digital media is an important maintaining factor of hikikomori: on the one hand, excessive online behavior will lead to less engagement in real-life social contacts and hence fuel withdrawal behavior, but on the other hand, once withdrawn, the internet provides an easy and accessible way to remain in some way connected to (people in) the external world [40]. Furthermore, hikikomori symptoms were positively correlated with social, coping, and conformity motives of going online, implying that higher levels of social withdrawal are associated with a stronger inclination to use the internet for getting in touch with others, dealing with stress and negative emotions, and avoiding the disapproval of others. Of these motives, only coping made a unique contribution to HQ-25 scores, indicating that – at least in this non-clinical sample – evading stress and adversity was the main reason for persons with strong hikikomori symptoms to use the internet. Although this aligns well with the observation that people who display high levels of problematic internet habits often go online to deal with problems in their life [80], it may well be that in clinical populations other motives of internet use are also implicated in hikikomori-like behavior.

Support was also found for the hypothesis that hikikomori symptoms would be higher in clinically referred participants with ASD or high levels of autistic traits than in non-clinical participants. On the HQ-25, 45.8% of the suspect ASD participants scored higher than the ‘cut-off’ for being at risk for hikikomori [48], which is close to the percentage of hikikomori as noted by Yamada et al. [27] in their sample of clinically referred ASD patients. These findings are in line with previous studies showing that there is a positive relationship between autism and autistic traits on the one hand and (symptoms of) the hikikomori syndrome on the other hand [23, 25, 26, 27]. A recent study has demonstrated that people high on the autism spectrum show a stronger tendency towards (extreme) social withdrawal because of the difficulties they experience in their social, emotional, and academic/occupational life [81]. Meanwhile, the possibility cannot be excluded that extreme social withdrawal is a radicalized pathognomonic symptom of ASD [45]. Whatever the case may be, it is clear that higher levels of hikikomori symptoms are oftentimes present in people suffering from this neurodevelopmental disorder.

A confirmatory factor analysis revealed that the expected three-correlated-factors model of the HQ-25 did not demonstrate a good fit in this relatively small Dutch sample. This is at odds with previous studies yielding support for the original three-factor solution for translated versions of the HQ-25 in a variety of countries such as Italy, Germany, France, Russia and Turkey [52, 53, 54, 55, 56, 57, 58]. As the translation was done according to the generally applicable standard, it seems most likely that sample characteristics accounted for this deviating finding. More precisely, our convenience sample was quite young and well-educated, and the data indeed showed that these demographic variables had some effect on participants’ HQ-25 scores. For example, age was found to be negatively correlated with hikikomori symptoms. The participants of this study were aged between 16 and 27 years and hence at some point in their developmental transition from adolescence to adulthood, during which youngsters gradually start to develop their own identity and attain more autonomy [82]. Social withdrawal is a normative phenomenon during this process but can become more extreme in those for whom this transition is difficult [45]. It is likely that that the older individuals in our sample had successfully taken this developmental hurdle, implying that the tendency to withdraw and seclude themselves were largely reduced. However, this does not mean that as development progresses, new life challenges may emerge, potentially triggering new episodes of social withdrawal, and in extreme cases, hikikomori [14, 83]. Furthermore, educational level also appeared negatively correlated with hikikomori symptoms. Thus, participants following lower levels of education reported higher levels of extreme social withdrawal. This is in keeping with findings of previous research [84] and underlines the importance of education as a context that may fuel marginalization of some individuals and hence promote the tendency towards social withdrawal [85]. Altogether, it seems important for future studies to test the factor structure of the Dutch translation of the HQ-25 in a larger, more representative sample, and to systematically explore differences in the experience of hikikomori symptoms across various age and educational groups.

An additional finding of this study was that the clinically referred sample of adolescents and young adults with suspect ASD reported higher levels of anthropophobia and paranoia in comparison with the non-clinical sample, which corroborates earlier observations that people with this neurodevelopmental disorder display higher levels of social anxiety disorder [86] and paranoid ideation [87]. Participants in the ASD sample also indicated to experience somewhat higher levels of loneliness in relation to peers but especially reported a stronger affinity for aloneness, which reflects that they may feel unhappy because their social needs are not fully met but at the same time also have a prevailing preference for solitude [88].

### Limitations

While this study provides valuable insights on hikikomori symptoms in a young Dutch population, it should also be acknowledged that the present study suffers from a number of limitations. First of all, the study was cross-sectional in nature, which means that we cannot draw conclusions on cause-effect relationships. In other words, with these data it was not possible to investigate whether psychopathological symptoms, loneliness/attitudes to aloneness, and (problematic) internet use are a cause or a consequence (or both) of extreme social withdrawal. Obviously, we need longitudinal research designs to establish the direction of the observed relations. A second shortcoming has to do with the fact that the study solely relied on self-report. For example, the inclusion of parent ratings of hikikomori symptoms and the other constructs would certainly have provided important cross-validational information. In relation to this, a cautionary note should be made because we used some of the self-report measures outside of their intended age range. This was specifically true for the LACA, which was developed for younger people, and the MUI, which so far has only been used in adult samples. Preferably, measures should be employed that have been validated across various age groups or simply restrict the study population to a specific developmental stage (i.e., either adolescence or adulthood). A third demerit pertains to the small sample size of the clinically referred sample (*n* = 24). It was also unfortunate that we did not include a scale to quantify autistic traits as this would have enabled us to explore the link between ASD and hikikomori symptoms in more detail. A fourth limitation was that the level of education of the participants in this study was quite high and hence not fully representative for the Dutch population. Future studies should try to include more participants following or having followed lower types of education. A final drawback was that the internet-related scales were not completed in the clinically referred sample. A recent meta-analytic review [89] has demonstrated that in particular (young) people with ASD are prone to develop problematic internet use, and so further exploration of this phenomenon and its potential connection to hikikomori symptoms in this population could be valuable [42].

### Conclusion

Hikikomori is an extreme form of social withdrawal that is increasingly seen in developed and industrialized Western and Eastern countries [12]. Hikikomori symptoms often manifest themselves during adolescence and young adulthood [90] and so it is important to learn more about this phenomenon and its psychopathological and psychosocial correlates in this period of life. In a convenience population of non-clinical and clinically referred Dutch participants aged 16 to 27 years, we found theoretically meaningful relations between hikikomori symptoms and anthropophobia (social anxiety), ASD, loneliness and an affinity for aloneness, and problematic internet use. All these variables appear to play a role in the pathogenesis of extreme social withdrawal, although they should be considered within a context of developmental transitions in a fast, agentic, and egocentric world in which some individuals increasingly run the risk to become marginalized [45].

## Data Availability

Data are available upon request from the corresponding author.
